# Betaine Mitigates Paclitaxel‐Induced Hepatotoxicity in Rats by Regulating Oxidative Stress, Inflammation, Apoptosis, and Autophagy

**DOI:** 10.1002/fsn3.71101

**Published:** 2025-10-16

**Authors:** Esmaeel Babaeenezhad, Niloofar Moammer, Marjan Joudaki, Omid Dezfoulian, Sahar Yarahmadi, Seifollah Bahramikia

**Affiliations:** ^1^ Nutritional Health Research Center Lorestan University of Medical Sciences Khorramabad Iran; ^2^ Department of Biochemistry and Genetics, School of Medicine Lorestan University of Medical Sciences Khorramabad Iran; ^3^ Department of Biology, Faculty of Basic Sciences Lorestan University Khorramabad Iran; ^4^ Department of Pathobiology, School of Veterinary Medicine Lorestan University Khorramabad Iran

**Keywords:** apoptosis, autophagy, betaine, hepatotoxicity, oxidative stress, paclitaxel

## Abstract

Paclitaxel (PTL) is widely used in chemotherapy; however, its efficacy is compromised by the risk of liver toxicity. This is the first study to investigate the protective effect of betaine (BTN) against PTL‐induced liver injury. Rats were randomly divided into five experimental groups (*n* = 7 per group): control (saline), BTN (100 mg/kg/day), PTL (2 mg/kg/day), PTL + BTN (2 and 50 mg/kg/day), and PTL + BTN (2 and 100 mg/kg/day). After the rats received 2 mg/kg body weight PTL intraperitoneally for the first five consecutive days, BTN was administered orally for 10 days. Our results indicate that BTN restores liver antioxidant levels (SOD, FRAP, and GSH), improves liver function by lowering AST and ALT levels, and attenuates PTL‐induced lipid peroxidation. In the livers of PTL‐treated rats, BTN significantly decreased the levels of NF‐κB and TNF‐α proteins and the ratio of caspase‐3/pro‐caspase‐3, whereas it increased the levels of Nrf2, LC3‐II/LC3‐I, and Bcl‐2. In summary, the results of this study suggest that BTN may ameliorate PTL‐induced liver injury by regulating oxidative stress, inflammation, apoptosis, and autophagy.

## Graphical abstract

This study investigated the protective effect of betaine (BTN) against paclitaxel (PTL)‐induced liver injury in rats. The results showed that betaine reduced PTL‐induced liver injury by improving antioxidant levels, reducing inflammation, apoptosis, and regulating autophagy.

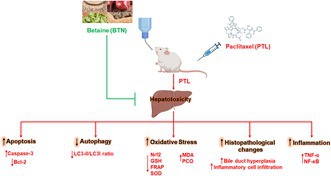

## 1 Introduction

Cancer represents a critical global health burden, with nearly 20 million new cases and 9.7 million deaths annually (Bray et al. 2024). This rising incidence emphasizes the urgent requirement for more effective therapeutic strategies than traditional modalities, including surgery, chemotherapy, radiotherapy, and immunotherapy (Abd‐Elrazek et al. 2020; DeVita and Chu 2008). Among current chemotherapeutic agents, paclitaxel (PTL), a member of the taxane family, is widely used for the treatment of lung, ovarian, and breast cancer (Gornstein and Schwarz 2014). The mechanism of action of PTL is to stabilize microtubules by binding to β‐tubulin subunits, preventing their normal depolymerization during mitosis (Barbuti and Chen 2015; Mandaliya et al. 2015). This leads to mitotic arrest in metaphase and ultimately triggers apoptosis in cancer cells (Gornstein and Schwarz 2014). Despite its clinical efficacy, the use of PTL is limited by its pronounced off‐target toxicity, particularly dose‐dependent hepatotoxicity (Yang et al. 2024), neurotoxicity (Gornstein and Schwarz 2014), nephrotoxicity (Gur et al. 2022), and testicular toxicity (Ileriturk et al. 2023). Minimizing these side effects is crucial for improving therapeutic outcomes and increasing patients' quality of life (Gornstein and Schwarz 2014).

Although PTL is widely recognized for its effectiveness in cancer therapy, its clinical application is significantly hampered by its dose‐dependent hepatotoxic effects (Yang et al. 2024). PTL exacerbates liver damage, elevates serum liver enzyme levels, and induces widespread hepatic necrosis, ultimately leading to death (Barbuti and Chen 2015). The precise molecular mechanisms underlying PTL‐induced hepatotoxicity remain incompletely understood; however, oxidative stress, inflammation, apoptosis, and autophagy have been implicated as major contributors (Ali et al. 2023; Çomaklı et al. 2023). Mechanistically, PTL enhances the production of reactive oxygen species (ROS) and induces mitochondrial dysfunction in hepatocytes by disrupting the Bax/Bcl‐2 ratio, thereby promoting apoptosis via cytochrome c release and caspase‐3 activation (Çomaklı et al. 2023). Furthermore, elevated ROS levels suppress endogenous antioxidant defense systems and initiate inflammatory responses by activating the NF‐κB signaling pathway (Ileriturk et al. 2023; Çomaklı et al. 2023). This activation facilitates the nuclear translocation of NF‐κB‐p65 and promotes the transcription of pro‐inflammatory cytokines, including TNF‐α and IL‐6 (Barbuti and Chen 2015).

Under conditions of severe cellular stress, such as nutrient deprivation or growth factor withdrawal, autophagy serves as a vital adaptive mechanism by selectively degrading damaged organelles, particularly dysfunctional mitochondria, thereby preventing mitochondria‐mediated cell death (Kim and Lemasters 2011; Mizushima and Komatsu 2011). This process is initiated by the formation of a double‐membrane structure known as an autophagosome, which encapsulates cytoplasmic components destined for degradation. Lysosomal enzymes destroy substances entrapped in autophagosome‐lysosome fusion (Kim and Lemasters 2011; Hailey et al. 2010). The conversion of the light chain of microtubule‐associated protein 1 (LC3‐I) from a cytosolic form to a lipidated, membrane‐bound form (LC3‐II), associated with the autophagosome membrane, serves as a popular biochemical indicator of autophagy (Kabeya et al. 2000). Several studies have shown that autophagy protects against drug‐induced liver injury (Chen et al. 2014). Autophagy selectively removes damaged mitochondria and proteins, thereby reducing ROS and pro‐apoptotic factors. In acetaminophen toxicity models, autophagy inhibition exacerbates liver injury, whereas its activation has a protective effect (Williams and Ding 2020). Similarly, in amiodarone‐induced hepatotoxicity, impaired autophagy is associated with increased hepatocyte apoptosis, emphasizing its protective role against drug‐induced endoplasmic reticulum stress and lipid accumulation (Brecklinghaus 2020). In addition, natural compounds such as curcumin and resveratrol exert hepatoprotective effects by stimulating autophagy (Zhao et al. 2023).

However, the current findings on the role of autophagy in this context remain inconsistent. While several studies have reported that autophagy activation confers hepatoprotective effects by reducing oxidative damage and promoting cellular homeostasis, conflicting evidence exists (Zhang et al. 2020; Wandrer et al. 2020). Notably, Gur et al. (2022) demonstrated that PTL induces autophagy in hepatocytes and exacerbates liver injury, suggesting that excessive or dysregulated autophagic activity may contribute to hepatotoxicity.

Based on these mechanisms, targeting oxidative stress, inflammation, and apoptosis and modulating autophagy may be an effective approach to mitigate PTL‐induced hepatotoxicity. Therefore, we hypothesized that the administration of betaine (BTN) could attenuate the hepatotoxic effects of PTL. BTN, also known as trimethylglycine, is found in spinach, sugar beets, and wholemeal products. BTN has been studied for its potent cytoprotective and antioxidant abilities in metabolic diseases, neurological disorders, and cancer, as well as its involvement in one‐carbon metabolism (Wang et al. 2010; Zhao et al. 2018). A study showed that the administration of 200 μM BTN to human L02 liver cells significantly enhanced Nrf2 nuclear translocation and the synthesis of antioxidant enzymes, including superoxide dismutase (SOD) and HO‐1, through an epigenetic process (Zhang et al. 2024). In a mouse model of acetaminophen‐induced acute hepatotoxicity, BTN increased the expression of Nrf2‐related cytoprotective genes, suggesting its antioxidant capability (Khodayar et al. 2020). The Nrf2 signaling pathway modulates nearly 3000 genes associated with detoxification, antioxidant protection, inflammation, and metabolism (McCord et al. 2023). Activation of this system by BTN increases the levels of endogenous antioxidants, such as glutathione (GSH), SOD, and catalase (CAT), which counteract ROS from sources such as PTL, providing comprehensive protection (Li et al. 2024; Galicia‐Moreno et al. 2020). When Nrf2 is activated, the function of NF‐κB is inhibited, leading to a reduction in inflammation and increased PTL toxicity (Li et al. 2024; Gao et al. 2022). Given that PTL‐induced hepatotoxicity is mediated by oxidative stress, inflammation, and apoptosis, targeting these mechanisms with BTN may offer a rational strategy for mitigating PTL‐related liver injury.

In addition to its anti‐inflammatory effect, BTN has also shown anti‐apoptotic properties (Veskovic et al. 2019). The upstream effects on reducing oxidative stress and inflammation, which promote the intrinsic apoptotic pathway, could explain this mechanism (Veskovic et al. 2019). Moreover, BTN modulates autophagy and attenuates hepatic fibrogenesis (Seo et al. 2024; Heidari et al. 2018). These multifunctional effects highlight BTN as a promising candidate for protection against PTL‐induced hepatotoxicity, a possibility that has not yet been systematically explored.

This study is the first to evaluate the potential hepatoprotective effects of BTN on PTL‐induced liver injury. Specifically, we investigated whether BTN alleviates PTL‐induced oxidative stress, inflammation, apoptosis, autophagy dysregulation, and histopathological alterations in the livers of rats. By elucidating these underlying mechanisms, our findings may support the development of complementary approaches to mitigate PTL‐associated hepatotoxicity and improve the therapeutic profile.

## 2 Material and Method

### 2.1 Chemicals

BTN was obtained from Sigma‐Aldrich Company (USA), and PTL was provided by Koçak Farma Company (Taksen 300 mg/50 mL, Turkey). Analytical‐grade chemicals, including 5,5′‐dithiobis (2‐nitrobenzoic acid) (DTNB), 2,4‐dinitrophenylhydrazine (DNPH), and guanidine hydrochloride, were purchased from Sigma‐Aldrich (USA). Additional reagents, such as ethanol, hydrogen peroxide (H_2_O_2_), thiobarbituric acid (TBA), trichloroacetic acid (TCA), and 5‐sulfosalicylic acid dihydrate, were procured from Merck (Darmstadt, Germany). The primary antibodies required for western blot analysis were obtained from Santa Cruz Biotechnology and Cell Signaling Technology (USA).

### 2.2 Experimental Design and Animal Grouping

To investigate the hepatoprotective effects of BTN against PTL‐induced liver injury, 35 adult male Wistar rats (180–220 g) were purchased from the Animal Lab of Kermanshah University of Medical Sciences (Kermanshah, Iran). All animals were maintained under controlled environmental conditions (23°C ± 2°C; 12‐h light/dark cycles) with unrestricted access to food and water. After an acclimation period of 1 week, the rats were randomly divided into five experimental groups (*n* = 7 per group). The groups were treated as follows:

1. Control group: Rats received intraperitoneal injections of normal saline (0.2 mL/day) for five consecutive days, followed by oral administration of normal saline (0.5 mL/day) for 10 days.
2. BTN 100 group: Animals received intraperitoneal injections of saline for 5 days, followed by oral gavage of BTN (100 mg/kg/day) for 10 days.
3. PTL group: Rats were treated with PTL (2 mg/kg/day, intraperitoneally) for 5 days, followed by oral administration of saline for 10 days.
4. PTL + BTN 50 group: Animals were administered PTL (2 mg/kg/day) intraperitoneally for 5 days and subsequently received BTN (50 mg/kg/day) via oral gavage for 10 days.
5. PTL + BTN 100 group: Similar to the previous group, PTL was administered intraperitoneally for 5 days, followed by a higher dose of BTN (100 mg/kg/day) for 10 days via oral gavage.

The selected doses of PTL and BTN were based on previously published studies confirming their pharmacological relevance and safety, namely, 2 mg/kg/day for PTL (Yakut et al. 2024) and 50 or 100 mg/kg/day for BTN administration (Huang et al. 2020; Zabrodina et al. 2016; Ahn et al. 2014).

At 24 h after the final gavage, the rats were anesthetized using ketamine (100 mg/kg) and xylazine (10 mg/kg) (Wellington et al. 2013), after which blood samples were collected, and the liver tissues were excised and divided into two equal parts. One part was immediately stored at −80°C for further biochemical evaluation. Another portion of the excised liver tissue was immediately fixed in 10% neutral‐buffered formalin for histopathological evaluation of the liver. Our study protocols were reviewed and approved by the Ethics Committee of Lorestan University of Medical Sciences (approval ID: IR.LUMS.REC.1403.345).

### 2.3 Histopathological Assessment

Liver tissues were carefully excised and fixed in 10% neutral‐buffered formalin for 72 h to preserve cellular architecture and morphology. Following fixation, the tissues were processed and embedded in paraffin blocks. Serial sections of 5 μm thickness were prepared and mounted on glass slides. These sections were stained with hematoxylin and eosin (H&E), a standard histopathological technique used to assess tissue structures and detect histopathological alterations (Yarahmadi et al. 2023).

### 2.4 Biochemical Evaluations

#### 2.4.1 Serum Activities of Aminotransferases

The activities of liver aminotransferases, including alanine aminotransferase (ALT) and aspartate aminotransferase (AST), were measured in the serum using commercially available kits (Pars Azmoon, Tehran, Iran).

#### 2.4.2 Oxidative Stress Markers

For the evaluation of oxidative stress biomarkers, liver tissues were homogenized (10% w/v) in ice‐cold phosphate‐buffered saline (PBS; 100 mM, pH 7.4). The prepared homogenates were centrifuged at 12,000×*g* for 20 min at 4°C. The resulting supernatant was carefully collected and stored at −80°C for subsequent analysis.

#### 2.4.3 Malondialdehyde (MDA) Concentration

Liver MDA levels, an established marker of lipid peroxidation, were quantified using the double heating method, as previously described (Draper and Hadley 1990). Briefly, 0.5 mL of the sample was mixed with 2.5 mL of 10% TCA and incubated in a boiling water bath (15 min). The cooled samples were centrifuged (3000×*g* for 10 min) to obtain a clear supernatant. Subsequently, 0.3 mL of the resulting supernatant was transferred to a new tube containing 0.3 mL of 0.67% TBA and heated at 95°C (20 min). The samples were cooled and subsequently analyzed spectrophotometrically at 532 nm. MDA concentrations were calculated using the molar extinction coefficient of the MDA‐TBA complex (*ε* = 1.56 × 10^5^ M^−1^cm^−1^) and expressed as μmol/mg of protein.

#### 2.4.4 Glutathione (GSH) Concentration

To evaluate GSH levels as an index of antioxidant capacity, we used the method established by Jollow et al. (1974). In this protocol, 0.5 mL of liver tissue homogenate was mixed with 1 mL of 4% sulfosalicylic acid and incubated at 4°C (1 h). Following centrifugation at 3000×*g* for 15 min, 1 mL of the supernatant was reacted with 0.1 mL of DTNB (4 mg/mL) and 0.1 M phosphate buffer (pH 7.4). The resulting yellow‐colored complex was quantified spectrophotometrically at 412 nm, and GSH concentrations were expressed as μmol/mg of protein.

#### 2.4.5 Ferric Reducing Ability of Plasma (FRAP) Assay

Samples (200 μL) were added to a prepared FRAP reagent containing acetate buffer (50 mL), ferric chloride (3 mL), and 2,4,6‐tripyridyl‐s‐triazine (TPTZ; 5 mL). The reaction mixture was incubated at 37°C (20 min) to enable the iron ions to be reduced by the antioxidants within the samples. Following incubation, the absorbance of the mixture was measured using a T80 UV/VIS spectrometer (UK) at 593 nm (Akbaribazm et al. 2020).

#### 2.4.6 Catalase (CAT) Activity

Liver CAT activity was assessed according to the method described by Aebi (1984). In this assay, 100 μL of supernatant was added to 1900 μL of 50 mM phosphate buffer (pH 7.0) in a quartz cuvette. The reaction was initiated by adding 1 mL of H_2_O_2_, and the decomposition rate of H_2_O_2_ was monitored spectrophotometrically at 240 nm using a T80 UV/VIS spectrometer (UK). Absorbance was recorded at 15, 30, 45, and 60 s, and CAT activity was reported as U/mg protein.

#### 2.4.7 Superoxide Dismutase (SOD) Activity

Liver SOD activity was measured using the nitro blue tetrazolium (NBT) reduction assay, which relies on the ability of the enzyme to inhibit the reduction of NBT (Harisa 2013; Beauchamp and Fridovich 1971). Briefly, the reaction mixture contained 2 mM riboflavin, 13 mM methionine, 75 mM NBT, and liver tissue supernatant. The absorbance was recorded at 560 nm, and SOD activity was expressed as U/mg protein.

#### 2.4.8 Protein Carbonyl (PCO) Levels

The PCO content was quantified using the method described by Reznick and Packer (1994). Briefly, 1 mL of 10 mM DNPH prepared in hydrochloric acid was added to 2 mg of protein sample. Following a 1 h incubation (25°C), 1 mL of 10% (w/v) TCA was added to precipitate proteins. The samples were then centrifuged (3000×*g*, 10 min), and the resulting pellets were washed thrice with 2 mL of a 1:1 ethanol–ethyl acetate mixture (v/v) to remove excess DNPH. The pellets were subsequently resuspended in 1 mL of 6 M guanidine hydrochloride (pH 2.3). The absorbance was measured at 370 nm after 10 min of incubation (25°C). PCO levels were determined using the DNPH extinction coefficient (*ε* = 2.2 × 10^4^ M^−1^cm^−1^) and expressed as nmol/mg protein.

### 2.5 Western Blotting

Western blotting was used to quantify the hepatic protein levels of Nrf2, NF‐κB‐p65, TNF‐α, Bcl‐2, pro‐caspase‐3, cleaved caspase‐3, LC3‐I, and LC3‐II. Liver tissues were homogenized in RIPA lysis buffer supplemented with protease inhibitors (SC‐24948), and the resulting lysates were centrifuged at 13,000 rpm for 10 min to obtain the soluble protein fraction. The total protein concentration was determined using the bicinchoninic acid (BCA) assay (Cat‐786‐570). Equal amounts of protein (30 μg per lane) were resolved by 8% SDS‐PAGE and subsequently transferred to polyvinylidene fluoride (PVDF) membranes. Membranes were blocked with 3% (w/v) skim milk in TBST for 3 h at room temperature and then incubated overnight at 4°C with the following primary antibodies: Nrf2 (1:1500, sc‐365,949), NF‐κB‐p65 (1:1000, sc‐8008), TNF‐α (1:1000, sc‐133,192), Bcl‐2 (1:1000, sc‐492), pro‐caspase‐3 (1:1000, sc‐271,759), cleaved caspase‐3 (1:1000, sc‐22,171‐R), LC3‐I/II (1:1000, cs‐2775), and β‐Actin (1:1000, sc‐517,582). Following primary incubation, the membranes were incubated with horseradish peroxidase‐conjugated secondary antibody (1:4000, sc‐2357) for 2 h. Protein bands were visualized using enhanced chemiluminescence (ECL, Cat‐1,705,061) and captured on X‐ray film. Densitometric analysis was performed using the ImageJ software (version 1.54 k), with β‐actin serving as the loading control.

### 2.6 Statistical Analysis

Statistical analyses were performed using GraphPad Prism software (version 8.0, USA). Data are presented as mean ± standard deviation (SD). One‐way analysis of variance (ANOVA) was employed to assess the overall differences among the experimental groups, followed by Tukey's post hoc test to determine specific intergroup comparisons. The Shapiro–Wilk test was used to assess the normality of the data. Statistical significance was set at *p* < 0.05.

## 3 Results

### 3.1 Effects of BTN on Hepatic Histopathological Changes in PTL‐Intoxicated Rats

**FIGURE 1:**
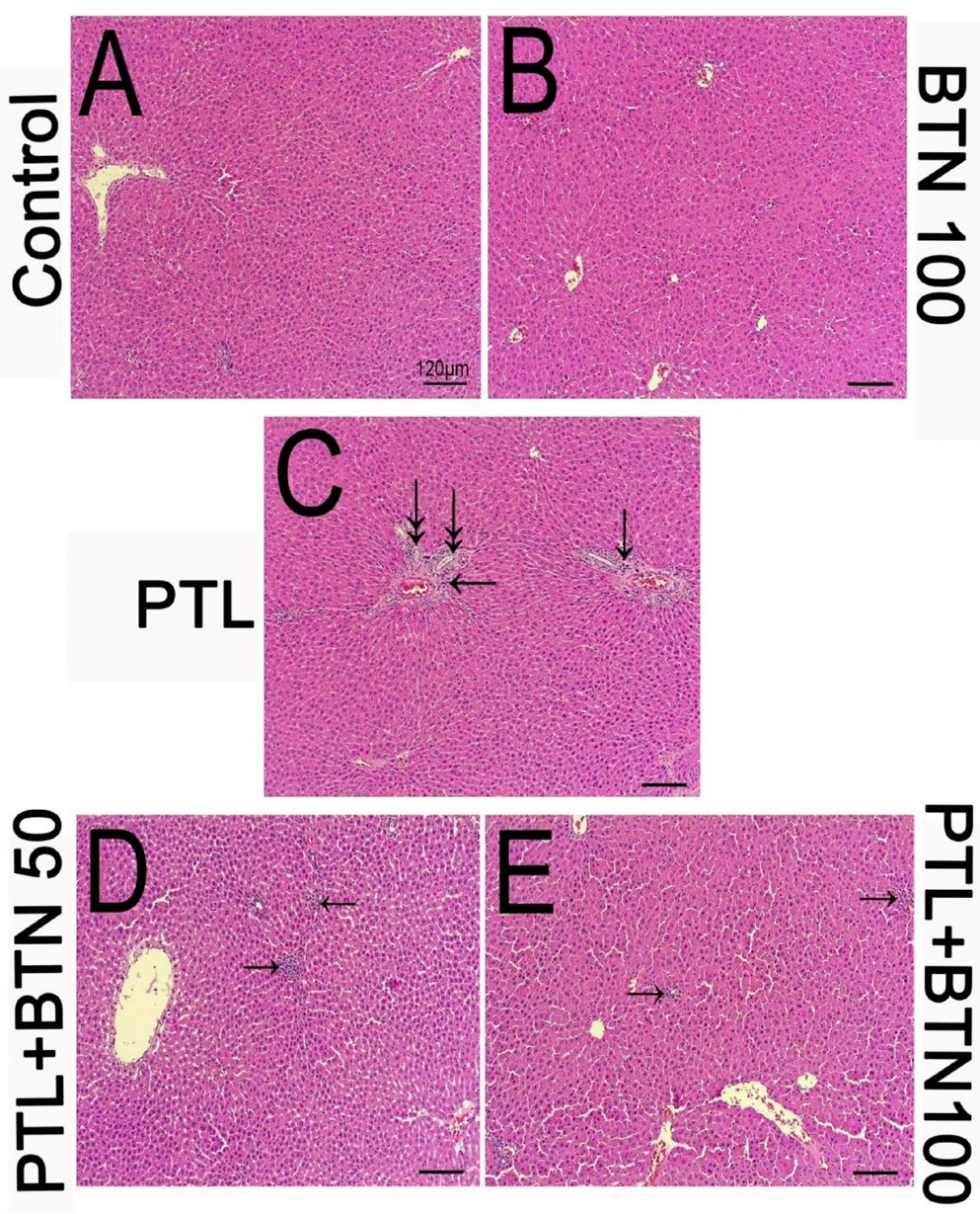
Effects of betaine (BTN) on paclitaxel (PTL)‐mediated hepatotoxicity in rats (A–E). Control group (A); normal hepatic architecture with well‐preserved hepatocytes and intact central veins. BTN 100 group (B); similar to the control group, shows no histological abnormalities, such as necrosis, fibrosis, inflammatory infiltration, or hyperplastic biliary ducts. PTL group (C); marked pericentrivenular fibrosis with infiltration of mononuclear inflammatory cells (arrows), prominent bile duct hyperplasia (double arrows), and mild necrosis are observed throughout the lesion. In addition, striking congestions are obvious in central veins. PTL + BTN 50 group (D); small focal aggregates of inflammatory cells are observed in the hepatic parenchyma. Biliary hyperplasia and fibrosis amounts are decreased (arrows). PTL + BTN 100 group (E); rare and sparse colonization of inflammatory cells with almost well‐stored hepatic architecture (arrows). Hematoxylin and eosin (H&E) staining; scale bar = 120 μm (×100 magnification) for all panels (A–E).

H&E‐stained liver sections from the control and BTN 100 groups revealed preserved hepatic architecture with no apparent histopathological abnormalities (Figure 1A,B). In contrast, liver tissues from the PTL group displayed notable pericentrivenular fibrosis, accompanied by the infiltration of mononuclear inflammatory cells scattered in the parenchyma (Figure 1C; arrows). In addition, bile duct hyperplasia was evident in the PTL group, as indicated by the presence of proliferating bile ducts (Figure 1C; double arrows). PTL‐intoxicated rats treated with 50 mg/kg BTN for 10 consecutive days exhibited a marked reduction in inflammatory cell infiltration compared to the PTL group. Histopathological examination revealed only mild focal aggregates of inflammatory cells within the hepatic parenchyma (Figure 1D; arrows), indicating partial attenuation of PTL‐induced liver injury by 50 mg/kg BTN. Interestingly, minimal inflammatory cell infiltration was observed in the hepatic tissue of PTL‐intoxicated rats treated with 100 mg/kg BTN (Figure 1E; arrows), indicating a more pronounced protective effect at the higher dose.

### 3.2 Effects of BTN on Serum Activities of Liver Aminotransferases in PTL‐Intoxicated Rats

**FIGURE 2:**
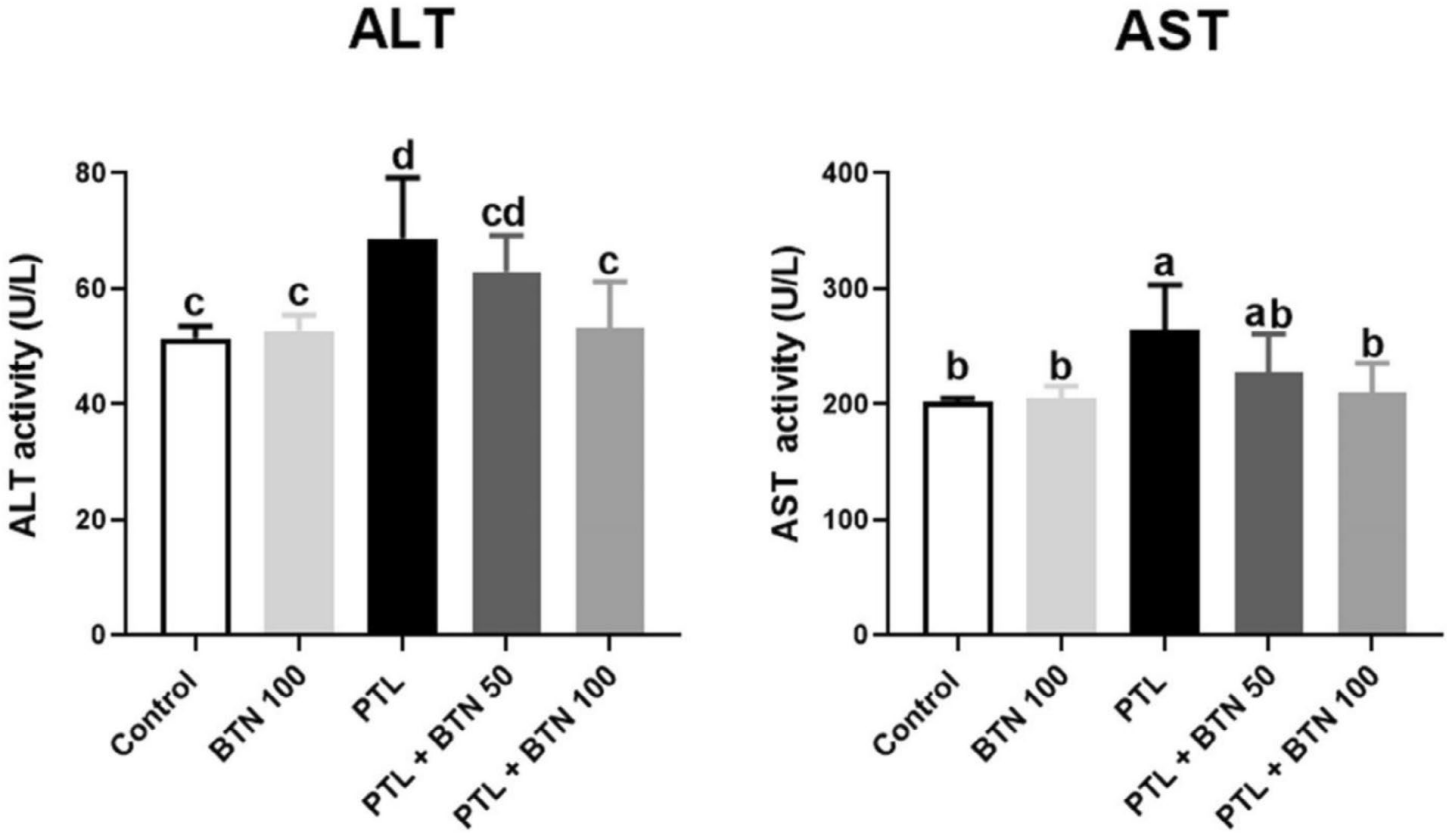
Effects of betaine (BTN) on the serum activities of aminotransferases (ALT and AST) in rats with paclitaxel (PTL)‐induced hepatotoxicity. BTN 100; betaine (100 mg/kg); PTL, paclitaxel (2 mg/kg); PTL + BTN 50/100; paclitaxel (2 mg/kg) + betaine (50/100 mg/kg). Bars labeled with different letters differ significantly (*p* < 0.05), whereas those sharing the same letter do not (*p* > 0.05).

Administration of PTL for five consecutive days resulted in a significant elevation of hepatic aminotransferase activities in the serum (Figure 2). Specifically, ALT and AST activities exhibited 1.33‐fold and 1.31‐fold increases, respectively, compared to those in the control group (*p* = 0.01 and 0.02, respectively). However, treatment with BTN for 10 consecutive days effectively mitigated these changes in a dose‐dependent manner. Administration of 50 mg/kg BTN led to a reduction in serum ALT and AST activities; however, these changes did not reach statistical significance (*p* > 0.05). Remarkably, administration of 100 mg/kg BTN significantly attenuated the PTL‐induced increase in ALT (22.54%) and AST (20.46%) activities (*p* = 0.03 and 0.04, respectively), restoring their levels to near those of the control group. These findings suggest that BTN may play a hepatoprotective role in PTL‐induced liver injury.

### 3.3 Effects of BTN on PTL‐Induced Oxidative Stress

Oxidative stress was assessed by quantifying MDA and PCO levels, evaluating GSH content and FRAP capacity, measuring the activities of key antioxidant enzymes, including SOD and CAT, and analyzing the expression of the upstream transcription factor Nrf2.

**FIGURE 3:**
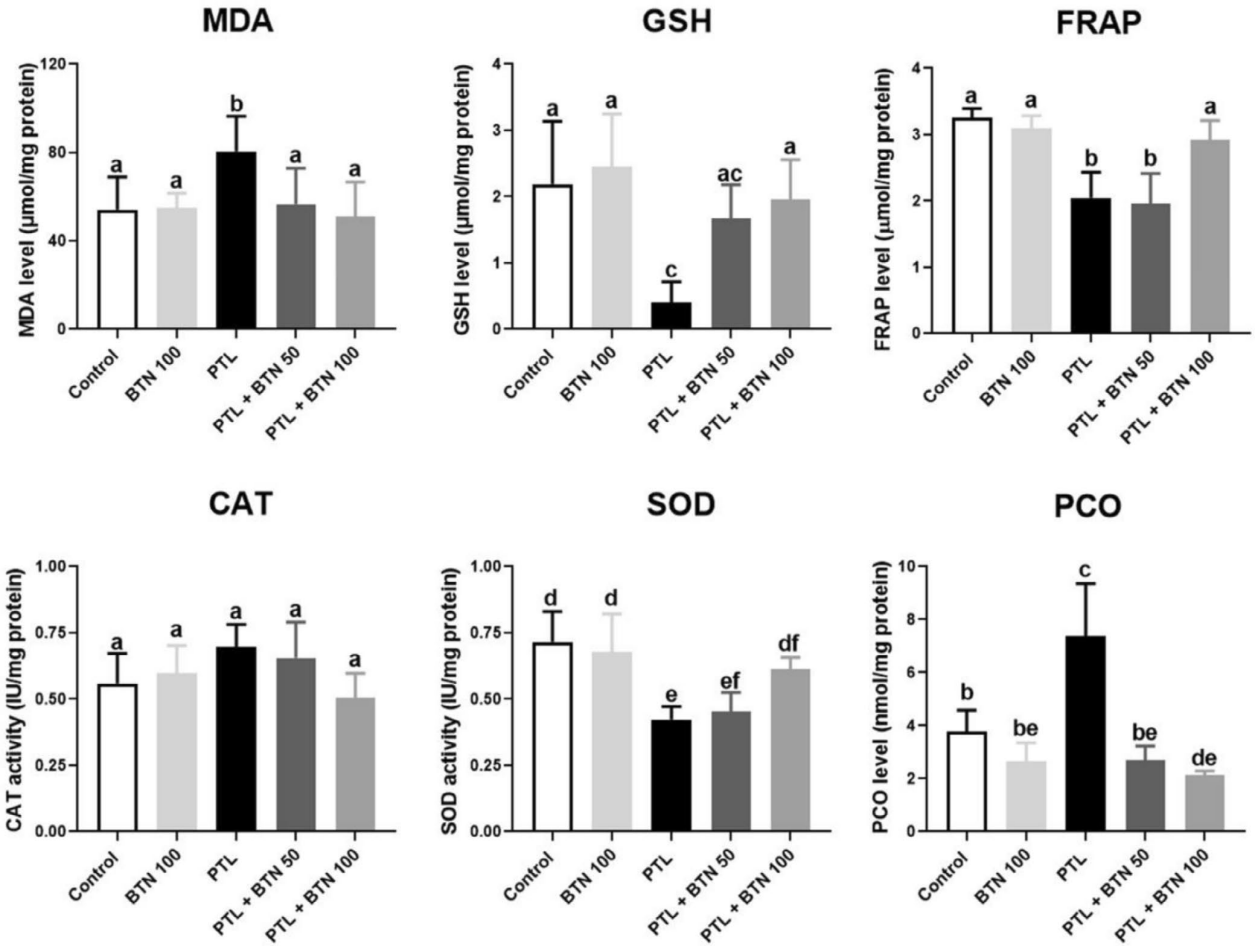
Effects of betaine (BTN) on oxidative stress biomarkers in rats with paclitaxel (PTL)‐induced hepatotoxicity. BTN 100; betaine (100 mg/kg); PTL, paclitaxel (2 mg/kg); PTL + BTN 50/100; paclitaxel (2 mg/kg) + betaine (50/100 mg/kg). Bars labeled with different letters differ significantly (*p* < 0.05), whereas those sharing the same letter do not (*p* > 0.05).

Administration of PTL for five consecutive days induced significant hepatotoxicity, as demonstrated by a marked increase in MDA and PCO levels, which increased approximately 1.49‐ and 1.96‐fold, respectively, compared to the control group (*p* = 0.03 and < 0.0001, respectively; Figure 3). Concurrently, GSH and FRAP levels showed a pronounced decline, decreasing by 5.41‐ and 1.58‐fold, respectively, compared with those in the control group (*p* = 0.0008 and 0.006, respectively; Figure 3). Treatment with 100 mg/kg BTN effectively mitigated these effects, significantly reducing MDA and PCO levels by 36.87% and 71.18%, respectively, in PTL‐intoxicated rats (*p* = 0.02 and < 0.0001, respectively; Figure 3). In contrast, PTL‐intoxicated rats receiving 100 mg/kg BTN had significantly increased GSH (4.82‐fold) and FRAP (1.42‐fold) levels compared with the PTL group (*p* = 0.01 and 0.04, respectively; Figure 3). BTN administration at 50 mg/kg exerted modest restorative effects on these oxidative stress markers; however, these changes were not statistically significant (*p* > 0.05; Figure 3). Among the evaluated parameters, only MDA and PCO levels were significantly reduced at this dose compared to the control group (*p* = 0.04 and < 0.0001, respectively; Figure 3), suggesting limited protective efficacy at the lower dose.

SOD activity was significantly suppressed in PTL‐treated rats, with SOD activity reduced by 1.69‐fold (*p* = 0.001) compared to that in the controls (Figure 3). In contrast, CAT activity increased by 1.24‐fold in the PTL group, although this change was not statistically significant (*p* = 0.36), possibly reflecting a compensatory response to oxidative stress (Figure 3). Liver SOD activity was significantly elevated (45.12%) in the PTL + BTN 100 group after receiving 100 mg/kg BTN compared with that in the PTL group (*p* = 0.04; Figure 3). BTN administration at 50 mg/kg failed to elicit a statistically significant improvement in SOD activity compared to the PTL‐treated group (*p* > 0.05; Figure 3), indicating an insufficient antioxidant response at this dose. Although CAT activity declined in the BTN‐treated groups, the reductions were not statistically significant (*p* > 0.05; Figure 3).

**FIGURE 4:**
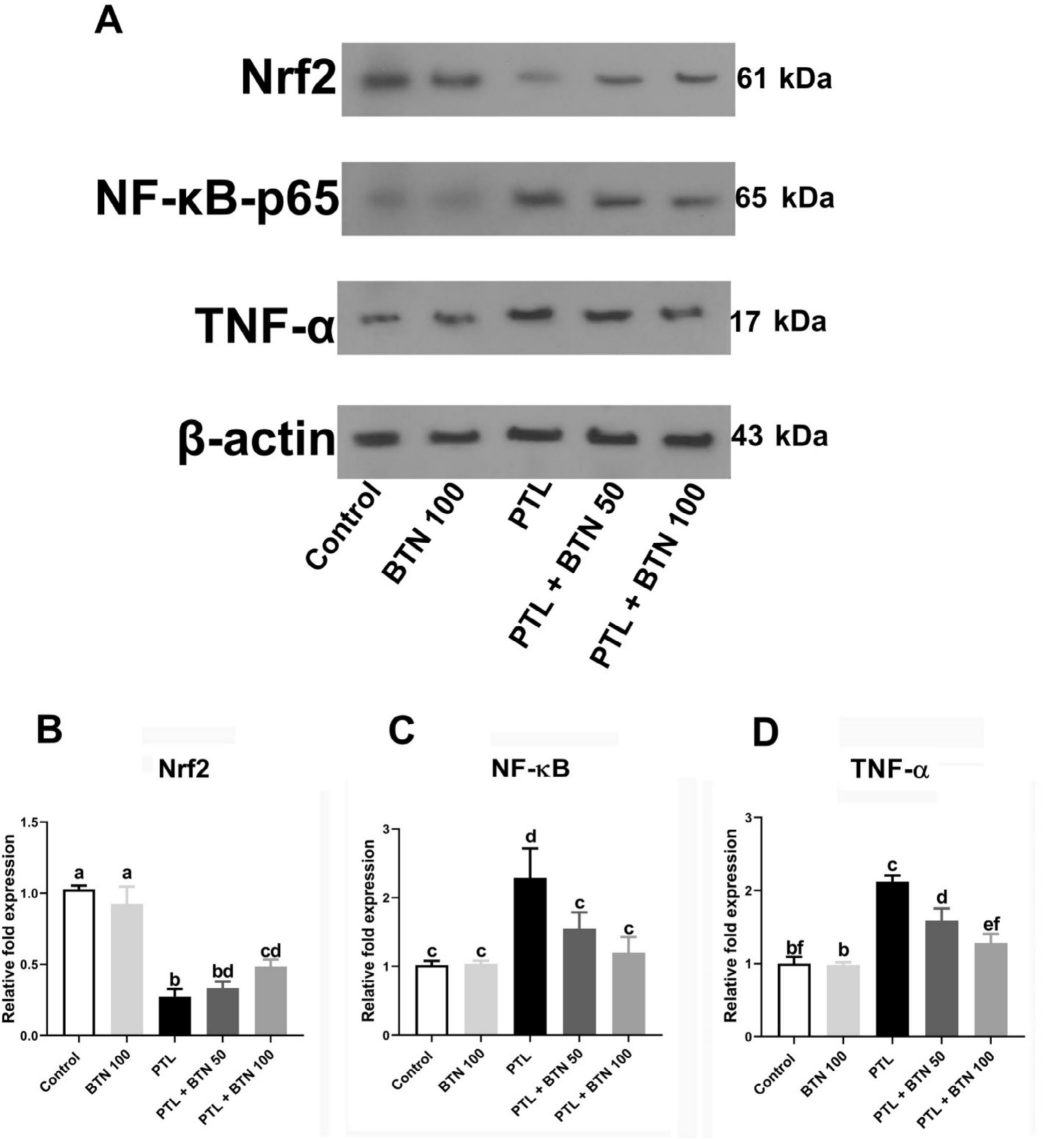
Effect of betaine (BTN) on Nrf2, NF‐κB, and TNF‐α protein expression in rats with paclitaxel (PTL)‐induced hepatotoxicity. Representative western blot bands (A), Densitometry analysis of Nrf2, NF‐κB and TNF‐α bands, respectively (B‐D). BTN 100; betaine (100 mg/kg); PTL, paclitaxel (2 mg/kg); PTL + BTN 50/100; paclitaxel (2 mg/kg) + betaine (50/100 mg/kg). Bars labeled with different letters differ significantly (*p* < 0.05), whereas those sharing the same letter do not (*p* > 0.05).

We determined the expression levels of the transcription factor Nrf2 in various groups using western blotting. After exposing rats to PTL for 5 days, Nrf2 expression was significantly downregulated by 3.76‐fold (*p* < 0.0001; Figure 4A,B). Interestingly, 100 mg/kg BTN administration considerably enhanced Nrf2 expression (77.48%) in the PTL + BTN 100 group compared with the PTL group (*p* < 0.0001; Figure 4A,B). Nrf2 expression was slightly increased following treatment with 50 mg/kg BTN compared to that in the PTL group, but the difference was not statistically significant (*p* > 0.05; Figure 4A,B).

### 3.4 Effects of BTN on PTL‐Induced Inflammation

Western blot analysis revealed that PTL administration elicited a robust pro‐inflammatory response, as evidenced by a significant upregulation in the expression levels of NF‐κB and TNF‐α proteins, showing 2.29‐fold and 2.12‐fold increases, respectively, compared to the control group (*p* = 0.0006 and < 0.0001, respectively; Figure 4A,C,D). Administration of BTN effectively attenuated these effects in a dose‐dependent manner. BTN at a lower dose (50 mg/kg) led to a significant decrease in NF‐κB and TNF‐α protein expression (1.47‐fold and 1.33‐fold, respectively) compared with that in the PTL group (*p* = 0.02 and 0.001, respectively; Figure 4A,C,D). Notably, in PTL‐intoxicated rats receiving 100 mg/kg BTN, NF‐κB and TNF‐α protein levels were significantly reduced by 1.90‐fold and 1.65‐fold, respectively, compared with those in the PTL group (*p* = 0.002 and < 0.0001, respectively; Figure 4A,C,D), indicating that BTN mitigates PTL‐induced hepatic inflammation.

### 3.5 Effects of BTN on Autophagic Biomarkers in PTL‐Treated Rats

**FIGURE 5:**
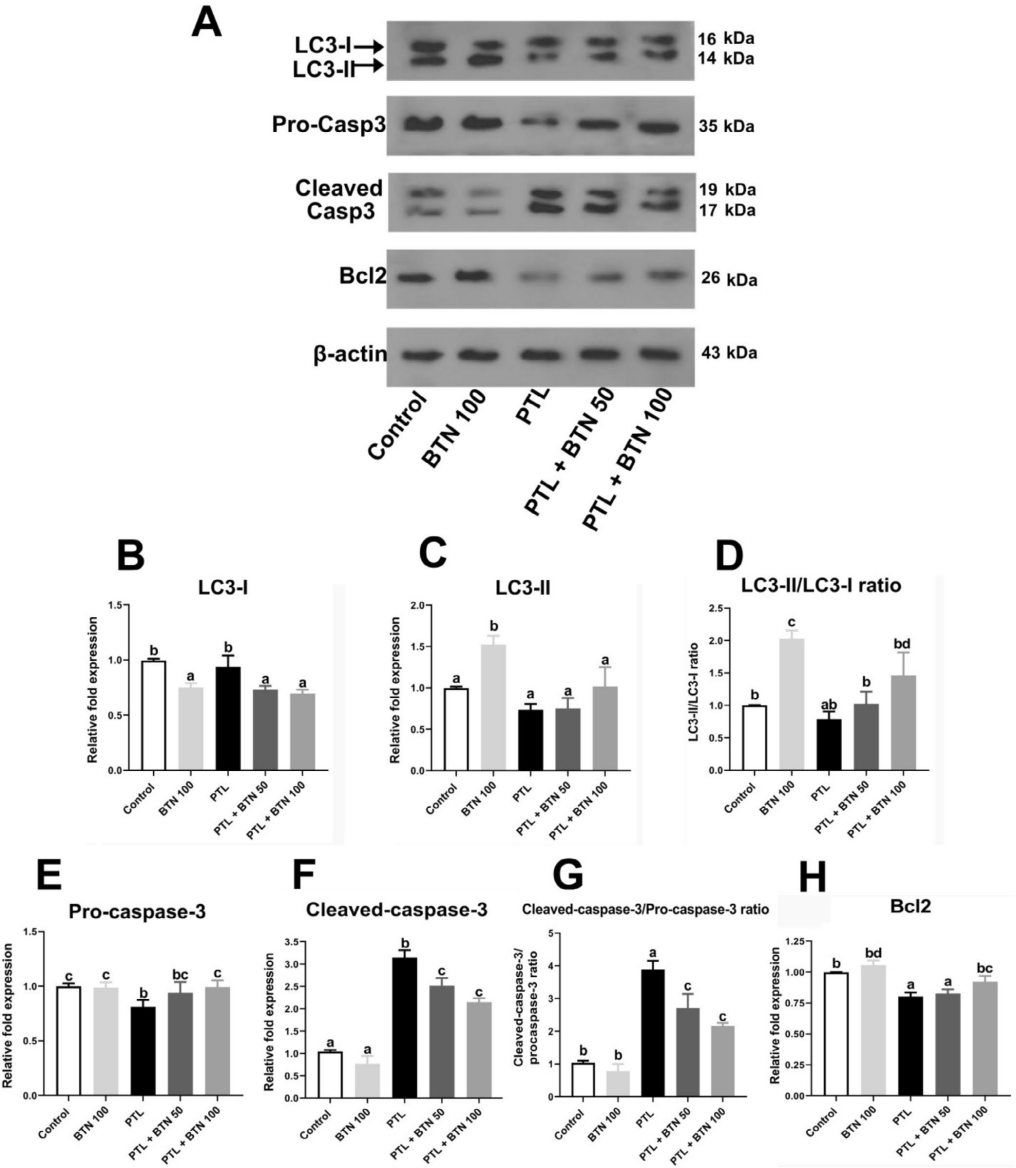
Effect of betaine (BTN) on liver tissue autophagic and apoptotic biomarkers in rats with paclitaxel (PTL)‐induced hepatotoxicity. Representative western blot bands (A), Densitometry analysis of LC3‐I, LC3‐II, LC3‐II/LC3‐I ratio, pro‐caspase‐3, cleaved‐caspase‐3, cleaved‐caspase‐3/pro‐caspase‐3, and Bcl2 bands, respectively (B‐H). BTN 100; betaine (100 mg/kg); PTL, paclitaxel (2 mg/kg); PTL + BTN 50/100; paclitaxel (2 mg/kg) + betaine (50/100 mg/kg). Bars labeled with different letters differ significantly (*p* < 0.05), whereas those sharing the same letter do not (*p* > 0.05).

We investigated the modulatory effects of BTN on autophagy‐related proteins LC3‐I and LC3‐II in rats with PTL‐induced liver injury. PTL administration slightly reduced LC3‐I and LC3‐II expression levels, as well as the LC3‐II/LC3‐I ratio in hepatic tissues compared to the control group; however, these changes were not statistically significant (*p* > 0.05; Figure 5A–D). Treatment with 50 and 100 mg/kg BTN significantly downregulated LC3‐I expression compared with the PTL group (*p* = 0.005 and 0.001, respectively; Figure 5A,B). Notably, BTN administration led to a significant increase in the LC3‐II/LC3‐I ratio, with the 100 mg/kg dose producing a 1.85‐fold increase compared with the PTL group (*p* = 0.01; Figure 5D), indicating enhanced autophagic flux. Although LC3‐II expression exhibited an upward trend following BTN treatment, these increases were not statistically significant (*p* = 0.14 and 0.17 for 50 and 100 mg/kg, respectively; Figure 5C).

### 3.6 Effects of BTN on Apoptotic Biomarkers in PTL‐Intoxicated Rats

Our findings demonstrated that exposure to PTL markedly shifted the hepatic cellular environment toward apoptosis, indicating its strong pro‐apoptotic potential. Specifically, PTL significantly decreased pro‐caspase‐3 expression (1.23‐fold) and markedly elevated cleaved caspase‐3 levels (3.02‐fold) and the cleaved/pro‐caspase‐3 ratio (3.73‐fold) compared to controls (*p* = 0.03, < 0.0001, and < 0.0001, respectively; Figure 5A,E–G). In parallel, a significant downregulation of the anti‐apoptotic marker Bcl2 was observed (1.24‐fold; *p* = 0.0003; Figure 5A,H), collectively confirming the activation of caspase‐dependent apoptosis following PTL exposure.

In contrast, BTN treatment effectively attenuated PTL‐induced apoptotic signaling. Administration of BTN at both doses (50 and 100 mg/kg) after PTL exposure significantly reduced the expression of cleaved caspase‐3 (*p* = 0.0018 and < 0.0001 for 50 and 100 mg/kg, respectively) and the cleaved/pro‐caspase‐3 ratio (*p* = 0.0012 and < 0.0001 for 50 and 100 mg/kg, respectively) compared with the PTL group (Figure 5A,F,G). Interestingly, a significant increase in pro‐caspase‐3 expression (1.22‐fold) was observed only at the 100 mg/kg dose of BTN compared with that in the PTL group (*p* = 0.04; Figure 5A,E). Furthermore, 100 mg/kg BTN significantly restored Bcl2 expression (*p* = 0.01; Figure 5A,H), with a 1.15‐fold increase relative to PTL‐treated rats. These data suggest that BTN confers hepatoprotection by modulating apoptotic signaling pathways, specifically through the inhibition of caspase‐3 activation and restoration of Bcl2‐mediated cell survival.

## 4 Discussion

This study provides significant evidence that BTN exhibits dose‐dependent hepatoprotective effects against PTL‐induced liver injury in rats. This study primarily demonstrates that high‐dose BTN (100 mg/kg) successfully alleviates multiple pathways of liver injury, including histopathological damage, aminotransferase elevation, oxidative stress, inflammation, and apoptosis. This study enhances our understanding of the hepatoprotective effects of BTN beyond its traditional role as a methyl donor by highlighting its ability to modulate autophagy and apoptotic signaling pathways.

The results showed that 5‐day PTL administration was sufficient to induce hepatotoxicity in rats, as evidenced by increased serum ALT and AST levels and histopathological changes in the liver tissue. The increase in serum ALT and AST activities may be due to hepatocyte damage, leading to an influx of enzymes into the bloodstream. Studies suggest that PTL can cause liver damage, which is reflected by elevated serum AST and ALT levels (Joerger et al. 2007; Gür and Bilgiç 2023). This study showed that 10‐day administration of BTN resulted in a significant improvement in PTL‐induced hepatotoxicity, such as decreased liver enzyme activity, consistent with previous studies showing the protective effects of betaine (Esfahani et al. 2023; Al‐Hafyan et al. 2024). According to a study by Al‐Hafyan et al. (2024) arsenite injection caused severe liver injury with elevated serum ALT and AST levels; however, treatment with BTN significantly reduced arsenite‐induced liver dysfunction. Another study showed that BTN significantly reduced the increase in liver enzymes caused by chloroform (Kim et al. 1998). Kim et al. (1998) found that lipopolysaccharide‐induced liver damage was confirmed by changes in ALT, ALP, and AST serum levels in the liver, whereas BTN or taurine supplementation significantly reduced the extent of these changes (Kim and Kim 2002). Treatment with BTN likely led to a decrease in serum AST and ALT levels by regenerating the histopathological changes in the liver of rats exposed to PTL, as documented for various natural antioxidants that exhibit hepatoprotective effects. The results of this study clearly demonstrate the hepatoprotective properties of BTN, consistent with the findings of Esfahani et al. (2023), who emphasized the beneficial effects of BTN on liver dysfunction. Moreover, this study, along with various in vivo studies (Ali et al. 2023; Yakut et al. 2024) has highlighted that PTL administration leads to oxidative stress, which plays a crucial role in the development of liver toxicity. Excessive production of ROS combined with impaired function of the body's antioxidant defense mechanisms leads to oxidative stress, which is a major contributor to cellular damage (Pieniążek et al. 2013; Demirci‐Çekiç et al. 2022). Overproduction of ROS has been associated with oxidative damage to several important macromolecules, including proteins, DNA, and lipids. Pathological conditions in the liver are associated with damage to these macromolecules (Demirci‐Çekiç et al. 2022; Juan et al. 2021).

As shown by the increase in MDA and PCO levels and the decrease in SOD, GSH, and FRAP levels, PTL significantly oxidized liver tissue in the current study. These adverse effects were reduced by BTN treatment. Only a few studies have investigated the hepatotoxicity of PTL. According to a study, PTL has a direct effect on the mitochondria and causes ROS formation in hepatocytes. This may be the cause of the significant decrease in antioxidant enzyme levels (Varbiro et al. 2001). Daily BTN administration significantly decreased lipid peroxidation and increased the levels of SOD, FRAP, and GSH in PTL‐treated rats. These results demonstrate the strong antioxidant effects of BTN, consistent with previous studies (Veskovic et al. 2019; Wang et al. 2021). Al‐Hafyan et al. (2024) showed that BTN increases liver levels of SOD, CAT, GPx, and GSH and simultaneously reduces MDA levels. In a model of non‐alcoholic fatty liver disease, BTN reduced ROS formation and strengthened the body's antioxidative defenses, leading to lower MDA levels and higher overall antioxidant capacity (particularly SOD, CAT, and GSH) (Veskovic et al. 2019). Moreover, Heidari et al. (2018) found that BTN (10–50 mg/kg) protects against acute (thioacetamide) and chronic (bile duct ligation) liver injury by improving mitochondrial function and reducing oxidative stress. Studies on isoniazid rifampicin‐induced hepatotoxicity showed that the protective effects of BTN were most pronounced at higher doses (250–500 mg/kg), with the optimal dose being 500 mg/kg (John et al. 2023).

The Nrf2/HO‐1‐signaling pathway is a frequently employed indicator for assessing oxidative stress (Gur et al. 2022; Zhou et al. 2020). Antioxidant levels, ameliorative phase II detoxification processes, and cellular redox balance are all regulated by the transcription factor Nrf2 (Lee and Johnson 2004; O'Connell and Hayes 2015). Khodayar et al. (2020) found that BTN pretreatment decreased Nrf2 expression. Chang et al. (2025) demonstrated that BTN induces the expression of genes encoding antioxidant enzymes during oxidative stress by upregulating nuclear Nrf2, a redox‐sensitive transcription factor that moves from the cytoplasm to the nucleus (Chang et al. 2025). Consistent with the above study, we demonstrated that Nrf2 expression increased by 77.48% following treatment with BTN 100 mg/kg, which aligns with this protective mechanism.

Oxidative damage can also cause inflammatory reactions by activating certain transcription factors, such as NF‐κB (Reuter et al. 2010). Inflammation caused by oxidative stress is associated with the abnormal production of inflammatory cytokines (TNF‐α, IL‐1, and IL‐6) and chemokines (IL‐8) (Reuter et al. 2010). Research conducted by Gur et al. (2022) revealed that PTL significantly increased the levels of IL‐1β, TNF‐α, and IL‐6, while enhancing the expression of NF‐κB mRNA transcripts. Their findings showed that hesperidin reduced the toxicities in liver tissues against inflammation and inhibited the activation of pro‐inflammatory genes by eliminating ROS produced from PTL. Unfortunately, there are insufficient studies on the effects of PTL on these processes in humans. In the present study, we found that the expression levels of the pro‐inflammatory cytokine TNF‐α and NF‐κB increased upon PTL treatment but decreased in a dose‐dependent manner upon BTN administration. The findings of this study are in line with previous research indicating that BTN possesses anti‐inflammatory properties by blocking the NF‐κB pathway in aging (Go et al. 2005), Alzheimer's disease, and neuroinflammation (Shi et al. 2019).

Oxidative stress and NF‐kB activation during PTL toxicity trigger the intrinsic apoptotic pathway (Çomaklı et al. 2023). PTL stabilizes tubulin dimers, inducing apoptosis and cell cycle arrest (Steinmetz and Prota 2018). Additionally, it has been shown that PTL activates Raf‐1, which phosphorylates Bcl‐2 (Wang et al. 2017). This process inactivates Bcl‐2 and prevents it from forming heterodimers with pro‐apoptotic Bax (Radha and Raghavan 2017), which increases the amount of free intracellular Bax and ultimately triggers the activation of caspases through the release of cytochrome c from the mitochondria, which is triggered by Bax. Bcl‐2 and Bcl‐xL reduce the apoptotic effects of PTL (Ferlini et al. 2003), whereas proteins that promote the opening of permeability transition pores allow PTL to induce programmed cell death in tumor cell lines (Khing et al. 2021). In line with previous studies, PTL increased caspase‐3 levels and decreased Bcl‐2 levels in liver tissue. BTN counteracted these effects, indicating that BTN exerts protective effects against PTL‐induced hepatotoxicity. Additionally, BTN significantly reduced caspase‐3 levels in response to LPS‐galactosamine (Rasineni et al. 2020), cisplatin (Hagar et al. 2019), and isoniazid‐rifampicin (John et al. 2023). The recurrence of Bcl‐2 expression (1.15‐fold increase) alongside diminished caspase‐3 activation implies the anti‐apoptotic actions of BTN in PTL‐induced liver injury.

As mentioned above, autophagy dysregulation plays a crucial role in drug‐induced hepatotoxicity (Chen et al. 2014). However, current evidence regarding the role of autophagy in this context is inconsistent. Although several studies have reported that autophagy activation has hepatoprotective effects by reducing oxidative damage and promoting cellular homeostasis (Zhang et al. 2020; Wandrer et al. 2020), there is conflicting evidence. In particular, Gur et al. (2022) demonstrated that PTL induces autophagy in hepatocytes and exacerbates liver injury, suggesting that excessive or dysregulated autophagy may contribute to hepatotoxicity. However, PTL‐induced spinal cord injury leads to a decrease in the expression of autophagy‐related genes, such as LC3A, LC3B, and Beclin‐1 (Yardım et al. 2021). In agreement with previous studies, PTL therapy reduced the protein levels of the LC3ll/LC3l ratio in our study. Seo et al. (2024) suggested that an important upstream mechanism for the prevention of steatosis, ER stress, and apoptosis by BTN in NAFLD is the activation of autophagy. They discovered that the beneficial effects of BTN were greatly diminished when this process was blocked by the autophagy inhibitor chloroquine, emphasizing the importance of autophagy in the hepatoprotective effects of BTN. BTN injection in this study increased the LC3ll/LC3l ratio, consistent with the results of Seo et al. (2024).

Despite the marked hepatoprotective effects of BTN, this study had some limitations. Although the sample size was sufficient for detecting major effects, it may limit generalization and statistical power for smaller effects. The 10‐day treatment period may not have captured the long‐term effects. Only male rats were used, which could limit the translation of the results to female rats and humans. Histopathological analysis relied on H&E staining, which may overlook subtle molecular changes. Advanced techniques, such as immunohistochemistry, electron microscopy, and omics analyses, could provide deeper mechanistic insights and are suggested for future studies.

Dose‐escalation studies are recommended to clarify the dose–response profile of BTN and identify the minimum effective dose. Long‐term studies are necessary to evaluate the potential side effects of BTN administration, particularly because of its critical role in methylation pathways (Rukkumani et al. 2004; Mukherjee 2020). Moreover, the role of mitophagy, a specialized form of autophagy that focuses on impaired mitochondria, necessitates an investigation into the effects of BTN on mitochondrial function (Lin et al. 2018; Masuda et al. 2019; Ding et al. 2019). Translational research, particularly phase I clinical trials in PTL‐treated patients, is required to evaluate safety and early efficacy outcomes. Finally, investigating combination therapies involving BTN and other hepatoprotective agents, such as silymarin and N‐acetylcysteine, may further enhance liver protection (Yang et al. 2024; Santacroce et al. 2023).

The ameliorative effects of BTN observed in this study indicate its potential applications beyond PTL‐induced liver injury. Through its multi‐pathway mechanisms, including the regulation of oxidative stress, inflammation, autophagy, and apoptosis, BTN may protect against hepatotoxicity caused by various chemotherapeutic agents, antimicrobials, and other hepatotoxic compounds, such as acetaminophen. The dose‐dependent response also provides a rationale for developing personalized hepatoprotective strategies based on individual risk factors and drug exposure (Mukherjee 2020; Vesković 2024).

Although the study design exhibited strong internal validity, generalization to human populations requires careful consideration. Differences in pharmacokinetics between rats and humans, as well as genetic variations affecting BTN metabolism, could affect its efficacy and optimal dosing in humans. The results are particularly relevant for patients with cancer receiving PTL‐based therapies (Mukherjee 2020) or individuals with pre‐existing liver conditions, who may benefit the most from preventive hepatoprotection (Yang et al. 2024).

Future research should focus on population‐based studies to define optimal dosing across diverse demographic groups, accounting for age, sex, genetic susceptibility, and concomitant medications. Developing predictive models that incorporate these variables may facilitate personalized hepatoprotective interventions and improve clinical outcomes (Zhang et al. 2016; Akanle et al. 2020). Additionally, translational studies using advanced mechanistic assays or omics approaches could further elucidate the protective mechanisms of BTN and support its clinical applications. Notably, the FDA has approved BTN as a safe supplement for homocystinuria treatment (Santacroce et al. 2023), indicating its convenience for consumption in clinical settings compared with other newly developed agents (Mukherjee 2020).

## 5 Conclusions

These findings indicate that BTN exerts hepatoprotective effects against PTL‐induced liver injury by attenuating oxidative stress, restoring liver function, and ameliorating histopathological damage. These protective effects appear to be mediated, at least in part, by the inhibition of the NF‐κB/TNF‐α inflammatory axis, enhancement of autophagic activity, and suppression of the intrinsic apoptotic signaling pathway. The antioxidant, anti‐inflammatory, and anti‐apoptotic properties of BTN, along with its ability to upregulate autophagy‐related biomarkers, collectively contribute to its beneficial role in mitigating PTL‐mediated hepatotoxicity. Nevertheless, further investigations are warranted to elucidate the underlying molecular mechanisms and validate the therapeutic potential of BTN in clinical settings.

## Author Contributions

E.B., S.B., and S.Y. conceived and designed the research. E.B., S.B., S.Y., N.M., O.D., and M.J. performed the experiments. E.B. and S.B. contributed new reagents or analytical tools. E.B. and S.Y. wrote the manuscript. All authors read and approved the manuscript. The authors declare that all data were generated in‐house and that no paper mill was used.

## Ethics Statement

All animal experiments were conducted in accordance with the principles of the Declaration of Helsinki. Animal experiments were performed in accordance with the ethical guidelines and regulations set by the Ministry of Health of Iran (ethical code: IR.LUMS.REC.1403.345, Research Number: 3949).

## Consent

The authors have nothing to report.

## Conflicts of Interest

The authors declare no conflicts of interest.

## Data Availability

The authors have nothing to report.
